# A meta-analysis for the effect of prophylactic GTN on the incidence of post-ERCP pancreatitis and on the successful rate of cannulation of bile ducts

**DOI:** 10.1186/1471-230X-10-85

**Published:** 2010-07-31

**Authors:** Bin Chen, Tao Fan, Chun-hui Wang

**Affiliations:** 1 Department of Gastroenterology, West China Hospital, Sichuan University, 37# Guoxue Road, Chengdu, 610041, China; 2 Department of Respiratory Medicine, West China Hospital, Sichuan University, 37# Guoxue Road, Chengdu, 610041, China

## Abstract

**Background:**

Glyceryl trinitrate (GTN) has been shown to be able to relax the sphincter of Oddi (SO) both in animals and humans. Theoretically, the use of these compounds during and after endoscopic retrograde cholangiopancreatgraphy (ERCP) could relax the biliary and pancreatic sphincters, facilitating cannulation of common bile duct (CBD) during the procedure, or minimizing potential pancreatic outflow obstruction after the procedure. However, clinical trials evaluating the protective effect of GTN on the post-endoscopic retrograde cholangiopancreatgraphy pancreatitis (PEP) have yielded inconclusive results. This meta-analysis is to systematically assess the effect of prophylactic administration of glyceryl trinitrate (GTN) on the prevention of PEP and the effect on the cannulation of bile ducts.

**Methods:**

By searching PubMed (1966 to September 2009), CENTRAL (Cochrane Controlled trials Register; issue 3, 2009) and EMBASE.com (1984 to September 2009), two independent reviewers systematically identified prospective randomized controlled trials (RCTs) detecting the effect of prophylactic GTN on the incidence of PEP and on the cannulation of bile ducts. A meta-analysis of these clinical trials was then performed.

**Results:**

There are 55/899(6.1%) patients suffering PEP in the treatment group versus 95/915(10.4%) patients in the placebo group. The overall pooled risk of PEP was significantly lower in the GTN group than in the placebo group (OR 0.56, 95% CI: 0.40 to 0.79, p = 0.001). Subgroup analyses suggested that GTN administered by the sublingual form (OR 0.34,95% CI:0.16 to 0.75, p = 0.007) is more effective than transdermal route(OR 0.64,95% CI:0.40 to 1.01, p = 0.05), and the protective effect of GTN was far more obvious in the centers with high incidence of PEP (OR 0.40, 95% CI:0.24 to 0.67, p = 0.0006) than those centers with a low incidence of PEP (OR 0.75, 95% CI: 0.47 to 1.20, p = 0.22). Additionally, the meta-analysis suggests that GTN was not helpful for the cannulation of bile ducts.

**Conclusion:**

We concluded that prophylactic administration of GTN may significantly reduce the incidence of PEP and not be helpful for the cannulation of bile ducts.

## Background

Endoscopic retrograde cholangiopancreatography (ERCP) is now well established in the diagnosis and treatment of pancreatobiliary disease. Pancreatitis remains one of the most feared complications of ERCP, with a frequency ranging from 1 to 40 per cent depending partly on the chosen definition of pancreatitis [1]. The pathogenesis of ERCP-induced pancreatitis has not been completely elucidated, but some risk factors were recognized as independent predictors, including younger age, female, pancreas divisum, sphincter of Oddi dysfunction, prior ERCP-induced pancreatitis, difficulty of cannulation, and pancreatic duct injection [2]. An obstructed outflow was also suggested as an important initiating step in developing post-ERCP pancreatitis [3].

Several mechanical and pharmacological interventions have been evaluated in the prevention of PEP. Prophylactic pancreatic stents have become standard of care for reducing PEP in high-risk cases. In contrast, trials of pharmacological therapy have generally yielded disappointing results [4]. So far, no pharmacological prophylaxis for PEP is widespread clinical use [5].

Glyceryl trinitrate (GTN) can reduce sphincter of Oddi pressure [6], an effect that has been used to facilitate endoscopic removal of medium- and small-sized stones from the biliary tract [7]. Theoretically, the use of these compounds during and after ERCP could relax the biliary and pancreatic sphincters, facilitating CBD cannulation during the procedure, or minimize potential pancreatic outflow obstruction after the procedure. Unlike the other potentially beneficial drug therapies, GTN has the advantage of being inexpensive and comparatively easily administered [3].

Despite these anticipated benefits, prospective clinical trials evaluating effect of GTN on the incidence of PEP and on the successful rate of cannulation of ducts have yielded inconclusive results. So we intended to perform a meta-analysis, aiming to evaluate whether prophylactic use of GTN can reduce the incidence of PEP and/or increase the successful rate of cannulation of ducts by systematically reviewing the published randomized therapeutic trials about this topic.

## Methods

### Literature Search

PubMed (1966 to September 2009), CENTRAL (Cochrane Controlled trials Register; issue 3, 2009) and EMBASE.com (1984 to September 2009) were searched by adopting the following strategy "(endoscopy* or ERCP* or endoscopic retrograde cholangiopancreatography* or pancreatitis* or PEP* or post-ERCP pancreatitis* or post-endoscopic retrograde cholangiopancreatography pancreatitis* or cannulation*) AND (GTN* or glyceryl trinitrate* or nitroglycerin*)". The results were limited to human studies and clinical trials without language limited. The manual searching of reference lists from potentially relevant papers was performed to identify any additional studies that may have been missed using the computer-assisted strategy. Before conducting this analysis, we have sought expert advice on ERCP and Evidence-based medicine, and they said that it is necessary to perform this analysis to help make clinical decision.

### Selection Criteria and Assessment

Two investigators independently reviewed titles and abstracts of all citations identified by the literature search. Potentially relevant studies were retrieved and selection criteria applied. The inclusive criteria were: (1) studies that examine the effect of GTN on the incidence of PEP and/or on the cannulation of ducts; (2) studies that were prospective, randomized and placebo controlled; (3) studies in humans; (4) date not duplicated in another manuscript; (5) the age of patient population should be over 18 years; (6) the patients were scheduled to undergo ERCP; (7) co-interventions (including treatment of complications) were allowed if administered equally to all intervention groups. The following exclusive selection criteria were set: (1) quasi-randomized trials and non-randomized studies; (2) the raw data was not completed; (3) repetitive reports; (4) difference of co-interventions between intervention arms. Eligible articles were reviewed and data were abstracted in a duplicated and independent manner by two investigators. Included studies were assessed for methodological quality in accordance with the Jadad composite scale. According to this scale, low quality studies had a score of ≤ 2 and high quality studies had a score of ≥ 3 [8].

### Statistical Analysis

If several trials were available for a specific topic, meta-analysis using the software Revman 4.2 (provided by the Cochrane Collaboration, Oxford, UK) with a fixed effects model and random effects model was performed. Statistical heterogeneity between trials was evaluated by the Cochran Chi-square test and defined at a P value less than 0.05. Pooled odds ratio (OR) was calculated using the general inverse variance (IV) fixed-effect model. If results were heterogeneous (p < 0.05), a random-effects model was employed. Pooled OR was presented as standard plots with 95 percent confidence intervals (CI). In case of clinical heterogeneity concerning study population and therapeutic modalities, pooling was not implemented and the results were assessed using subgroup analyses or descriptive statistics.

## Result

**Figure 1:**
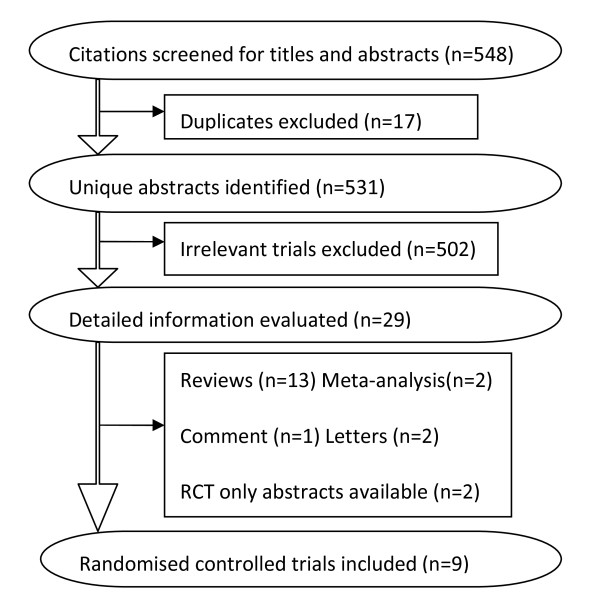
**Flow of study identification, inclusion, exclusion**.

Figure 1 shows details of study identification, inclusion, and exclusion. The search on PubMed, Embase and the Cochrane Library under the defined terms yielded 548 articles. Of these, we included 9 unique studies in this meta-analysis. Additionally, there are two articles without full-text available [9,10]. So seven randomized controlled clinical trials were included for the meta-analysis of the effect of GTN on the prevention of PEP [1,3,11-15] and seven RCTs were included for the meta-analysis of the effect of GTN on the cannulation of bile ducts [1,3,11-13,16,17]. Characteristics of the 9 included randomized controlled trials (RCTs) are listed in Table 1. Quality assessment by Jadad score revealed that five included studies met 5/5 criteria, and four studies met 4/5 criteria (Table 2), indicating that all studies were of reasonable methodological and high quality; none of the studies had any "fatal" methodological flaws. It is worth mentioning that a study made by Wehrmann T et al was also included in the meta-analysis of the effect of GTN on the incidence of PEP. Although its title and abstract didn't mention the outcome of PEP, yet there was a report for the incidence of PEP in its results [12].

**Table 1:** Characteristics of RCTs

First auther	Year	Location	Sample size	Intervention(treatment/control)	Related outcomes	Allocation concealment
Sudhindran S	2001	UK	186	2 mg sublingual GTN 90/96 Control	A and B	ADEQUAT

Wehrmann T	2001	Germany	80	10 mg topical GTN 40/40 Control	A and B	NOT CLEAR

Ghori A	2002	UK	254	0.4-0.8 mg sublingualGTN128/126 Control	B	ADEQUATE

Moretó M	2003	Spain	144	15 mg transdermal GTN 71/73 Control	A and B	ADEQUATE

Talwar A	2005	UK	104	5 mg topical GTN52/52 Control	B	NOT CLEAR

Kaffes AJ	2006	Australia	318	5 mg transdermal GTN 155/163 Control	A and B	NOT CLEAR

Beauchant M	2008	France	208	< mg intravenous GTN 105/103 Control	A and B	NOT CLEAR

Nøjgaard C	2009	Norway	806	15 mg transdermal GTN 401/405 Control	A and B	ADEQUATE

Hao JY	2009	China	74	5 mg sublingual GTN 38/36 Control	A	NOT CLEAR

A: the outcome of PEP			B:the outcome of cannulation			

**The definition of the post-ERCP pancreatitis**						

Sudhindran S	Acute pancreatitis was defined as a serum amylase level greater than 1000 (normal range 5-300) units/ml at 6 h in association with a visual analogue pain score of more than 5.					

Wehrmann T	Pancreatitis, defined according to published recommendations by Cotton PB et al (1991).					

Moretó M	Pancreatitis, defined according to published recommendations by Cotton PB et al (1991).					

Kaffes AJ	Pancreatitis, defined according to published recommendations by Cotton PB et al (1991).					

Beauchant M	Pancreatitis, defined according to published recommendations by Cotton PB et al (1991).					

Nøjgaard C	Pancreatitis, defined according to published recommendations by Cotton PB et al (1991).					

Hao JY	Post-ERCP pancreatitis could be defined as a disease with sustained pancreatitis symptoms (such as abdominal pain) and high-amylase value over the normal value after ERCP.					

**Table 2:** Jadad quality scores of randomized controlled trials included in the meta-analysis

Author	Randomization	Blind	Withdrawals and dropouts	Jadad score	setting
Sudhindran S	CGL	double	clear reported	5	single center

Wehrmann T	not clear	double	clear reported	4	single center

Ghori A	not clear	double	clear reported	4	single center

Moretó M	not clear	double	clear reported	4	single center

Talwar A	CGL	double	clear reported	5	single center

Kaffes AJ	CGL	double	clear reported	5	single center

Beauchant M	centrally	double	clear reported	5	multicenter

Nøjgaard C	adequate	double	clear reported	5	multicenter

Hao JY	not clear	double	clear reported	4	single center


CGL: computer-generated list

### Meta-analysis for the effect of GTN on the incidence of PEP

Seven studies were finally included in the meta-analysis for the effect of GTN on the incidence of PEP. In total, the analysis comprised 1841 patients with 150 patients suffering PEP. Among PEP-suffering patients, 55 patients were treated with GTN, whereas 95 patients were treated with placebo. The route of GTN administration differs remarkably in the trials and we conducted a subgroup meta-analysis looking at the trials where GTN was administered by transdermal patch or by sublingual route. The chi-square test for heterogeneity on all seven studies was 6.09 (df = 6, P = 0.41), i.e. no evidence of statistical heterogeneity was demonstrated and a fixed effects analysis model was performed.

**Figure 2:**
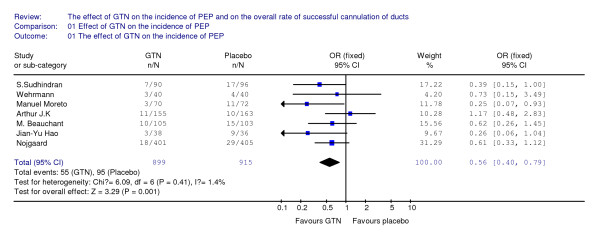
**This figure revealed the overall effect of GTN on the incidence of PEP, when all of seven included RCTs are analyzed together**.

The meta-analysis detected a significant difference between the two arms with regard to the incidence of post-ERCP pancreatitis (OR 0.56, 95%CI: 0.40 to 0.79, p = 0.001) (Figure 2), showing that the risk of pancreatitis was significantly lower in the treatment group than in the placebo group.

Because of the different ways of administrating GTN that could influence the effectiveness of GTN, we performed some subgroup meta-analysis of these trials (Table 3). There are two articles regarding the sublingual route in which there are 10/128 (7.8%) patients suffering PEP in the treatment arm versus 26/132 (19.7%) in the placebo arm; and there are three studies with regard to the transdermal route in which there are 32/626 (5.1%) patients suffering PEP in the treatment arm versus versus 50/640 (7.8%) in the placebo arm. When just analyzing the effect of sublingual GTN on the incidence of PEP, we revealed a significant difference between the two groups (OR 0.34, 95% CI: 0.16 to 0.75, P = 0.007), but there was no statistical difference found between the two groups when we just analyzed the effect of transdermal GTN on the incidence of PEP (OR 0.64, 95% CI: 0.40 to 1.01, P = 0.05). And so if we put the two results together, we could easily conclude that the way of sublingual GTN is more effective than the way of transdermal GTN for preventing the PEP, although the later enrolled far more patients (sublingual GTN 260 VS transdermal GTN 1266). The chi-square test for heterogeneity of the two subgroup analysis was P = 0.16 and P = 0.15, respectively, demonstrating no evidence of statistical heterogeneity. We suggested that investigation regarding the sublingual form for preventing PEP should be paid more attention to, and more RCTs should be per-formed to further confirm the effect of sublingual form on PEP.

**Table 3:** subgroup and sensitivity analysis of the effect of GTN on the prevention of PEP

Trials	Subgroup(N)	Odds ratio (95%) CI	Z	P value	Heterogeneity		
					**x^2^**	**P**	**I^2^,%**

**The overall effect of GTN on the incidence of PEP**							

all forms	7 studies(n = 1814)	0.56 [0.40, 0.79]	3.29	0.001	6.09	0.41	1.4

**Different forms**							

sublingual form	2 studies(n = 260)	0.34 [0.16, 0.75]	2.70	0.007	0.24	0.62	0

transdermal form	3 studies(n = 1266)	0.64 [0.40, 1.01]	1.93	0.05	3.77	0.15	47.0

**Different definition of PEP**							

the same criteria	5 studies(n = 1554)	0.64 [0.43, 0.94]	2.25	0.02	3.80	0.43	0

Sudhindran S	1 studies(n = 186)	0.39 [0.15, 1.00]					

Hao JY	1 studies(n = 74)	0.26 [0.06, 1.04]					

**Different incidence of PEP in the placebo group(10.4% as the cut-off point to stratify the trials)**							

low incidence	3 studies(n = 1204)	0.75 [0.47, 1.20]	1.22	0.22	1.42	0.49	0

high incidence	4 studies(n = 610)	0.40 [0.24, 0.67]	3.44	0.0006	1.88	0.60	0

Given the different definition of PEP may influence the pooled effect, we performed a sensitivity analysis including the five studies with the same definition of PEP derived from the published recommendations by Cotton PB et al [18], also detecting a significant protective effect of GTN against post-ERCP pancreatitis (OR 0.64, 95% CI: 0.43 to 0.94, P = 0.02). Through another subgroup analysis, we discover that prophylactic use of GTN before ERCP was very effectively against the occurrence of PEP in those centers with high incidence of PEP in the placebo arm (OR 0.40, 95% CI: 0.24 to 0.67, P = 0.0006). In contrast, prophylactic use of GTN before ERCP was not helpful in the prevention of PEP in those centers with low incidence of PEP (OR 0.75 95% CI: 0.47 to 1.20, P = 0.22). Because the overall incidence of PEP in the placebo group was 10.4%, so we adopted a decision that take 10.4% as the cut-off point to stratify the trials (see Table 3).

### Meta-analysis for the effect of GTN on the cannulation of ducts

Seven studies were included in the meta-analysis of GTN on the cannulation of bile ducts. Although not all of the seven studies considered the successful rate of cannulation as the major outcome, they all included information about it. We respectively pooled the primary successful rate of cannulation of ducts before the operation of sphincterotomy or pre-cut maneuver and the overall successful rate of cannulation of ducts after the operation of sphincterotomy or pre-cut maneuver. At first, we must make it clear that although some articles don't directly report the primary number of successful cannulation, but we can get the number through a subtraction that the overall number of successful cannulation subtracts the number of pre-cut maneuver and/or sphincterotomy and if we finally can't obtain the primary number of successful cannulation, thus we excluded these articles. All of seven included studies adequately report the overall successful number of cannulation and the number was obtained from just five included articles.

**Figure 3:**
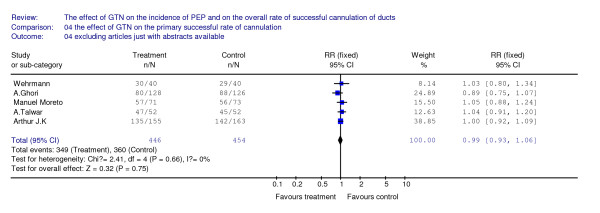
**This figure revealed the primary effect of GTN on the successful rate of cannulation**.

**Figure 4:**
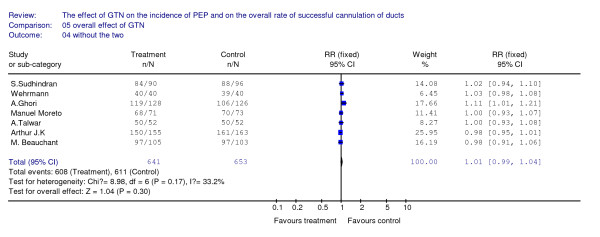
**This figure revealed the overall effect of GTN on the successful rate of cannulation**.

As for either the primary rate or the overall rate of successful cannulation, there isn't any benefit detected between the treatment arm and the placebo arm. There are five articles included with 900 patients for analyzing the effect of GTN on the primary rate of cannulation, and we don't detect a significant difference (see Figure 3). There are seven articles included with 1294 patients for analyzing the effect of GTN on the overall rate of cannulation, we also can't found any benefit from the use of GTN (see Figure 4).

When we added the two articles [9,10] only with abstract available to the seven included studies to analyze together and make some subgroup analysis, but it was a pity that as we have seen in the Table 4, there still was no benefit revealed for the cannulation. From all these analysis above, we can make a conclusion that the prophylactic use of GTN before ERCP may not be able to help facilitate cannulation of bile ducts and reduce the use of pre-cut maneuver.

**Table 4:** Meta-analysis of all seven included RCTs together with two articles only with abstract

Trials	Subgroup(N)	Risk ratio (95%) CI	Z	P value	Heterogeneity		
					**x^2^**	**P**	**I^2^,%**

**The effect of GTN on the primary rate of cannulation**							

all forms^#^	7 studies(n = 1233)	1.02 [0.96, 1.07]	0.57	0.57	6.22	0.40	3.6

all forms*	5 studies(n = 900)	0.99 [0.93, 1.06]	0.32	0.75	2.41	0.66	0

sublingual form^#^	3 studies(n = 587)	1.01 [0.93, 1.09]	0.22	0.83	6.12	0.05	67.3

topical form	2 studies(n = 184)	1.04 [0.91, 1.19]	0.59	0.56	0.00	0.95	0

**The effect of GTN on the overall rate of cannulation**							

all forms^#^	8 studies(n = 1376)	1.01 [0.99, 1.04]	1.01	0.31	8.87	0.26	21.1

all forms*	7 studies(n = 1294)	1.01 [0.99, 1.04]	1.04	0.30	8.98	0.17	32.3

sublingual form^#^	3 studies(n = 552)	1.06 [0.98, 1.15]	1.95	0.05	2.91	0.23	31.3

topical form	2 studies(n = 184)	1.01 [0.96, 1.06]	0.45	0.65	0.39	0.53	0

^#^: analysis with one or both of the two articles only with abstract available

*: analysis without the two articles only with abstract available

### Adverse effect of using GTN

Sudhindran S, et al reported that the only significant adverse effects attributable to GTN were hypotension and headache. More than 50% of the treatment group suffered a period of hypotension (systolic brachial pressure lower than 100 mmHg) in the immediate post-procedural period, compared with 5% of the placebo group. However, the hypotension responded in all cases to an intravenous infusion of crystalloids. And there are a small number of patients in the treatment group complained of headache.

Moretó M, et al found that both headache and hypotension are more often occurred in the treatment group than in the placebo group, and responded to conventional treatment. In addition, there are several patients having nausea and/or vomiting. Arthur JK, et al also found headache and hypotension are more frequently seen in the treatment arm. And the side effect of rash occurred in this study.

Beauchant M, et al and Nøjgaard C, et al also found headache and hypotension are more frequent in the GTN group. However, Ghori A, et al and Talwar a, et al revealed no adverse effect attributable to GTN in their studies.

In aggregate, hypotension and headache often occur and are more frequent seen in the GTN group. However, they easily managed and responded to conventional treatment. Additionally, other relatively less symptoms include nausea, vomiting and rash. So the side effect doesn't make an obstacle for the appropriate and widespread use of GTN.

## Discussion

Our present meta-analysis of RCTs evaluating the protective effect of GTN on PEP and the cannulaiton of ducts shows that the use of GTN before procedure was effective just in the prevention of PEP. Although the sublingual route seems to be more effective than the transdermal form, yet the number of those enrolled patients in the former wasn't enough (260 patients) compared with the latter (1266 patients) and thus perhaps made it not convincing. More importantly, we found that it is mainly for the centers with high incidence of post-ERCP pancreatitis that the pro-tective role of GTN against PEP was exerted rather than for the centers with low incidence of PEP, which is especially of practical meaning for those centers without experienced endoscopists and/or advanced instruments.

Hitherto, the etiology of ERCP-induced pancreatitis is not completely understood. The most commonly held, but largely unproven, hypothesis is that the injury initiating pancreatitis is predominantly mechanical, resulting from cannulation-induced spasm of the sphincter of Oddi and consequent temporary pancreatic duct obstruction [1].

It was demonstrated that Glyceryl trinitrate (GTN), a nitric oxide donor, lowered basal pressure and contraction amplitude in the sphincter of Oddi [6], an effect that has been used to facilitate endoscopic removal of medium- and small-sized stones from the biliary tract [7]. In selected difficult cases GTN is occasionally administered sublingually by some endoscopists to facilitate cannulation [8].

But our analysis suggested that prophylactic use of GTN before ERCP seems not to be helpful for increasing the successful rate cannulation of bile ducts. Furthermore, it can't be able to help reduce the attempts of cannulation, the time of procedure, or even the use of pre-cut especially for topical and transdermal route. Wehrmann T et al [12] reported that neither the number of cannulation attempts nor the time until successful cannulation differed significantly between the groups and the pre-cut rate was nearly identical in the two groups. Talwar A et al [17], topically administered GTN to the sphincter of Oddi did not aid in obtaining a cholangiogram or cannulation during an ERCP. However, the sublingual form of GTN seems to be relatively effective in increasing the successful rate of cannulation.

Why does the sublingual form appear to be more effective than the other forms? It seems that this phenomenon can't be explained clearly according to the currently existing information.

There are some other agents reported for prophylactic use before ERCP to reduce the incidence of PEP. Of these, it was demonstrated that NSAIDs was effective in the preventing PEP [5,19,20] and somatostatin and gabexate seems not to be effective [21,22]. Badalov N et al [4] reviewed the prophylactic using of all kinds of agents. Firstly, regarding to drugs that decrease inflammation, there was just NSAIDs showing clear benefits, with other agents such as allopurinol, n-acetylcysteineIn, corticosteroids and interleukin-10 (IL-10) showing no clear benefits. Secondly, for drugs that interrupt the activity of proteases including heparin, gabexate maleate and ulinastatin, there are still no clear benefits detected. Finally, inhibitors of pancreatic secretion including somatostatin, calcitonin and glucagon showed no protective effect for PEP, yet maybe octreotide and beta-carotine administration had a beneficial effect. Additionally, pancreatic stent placement and guidewire cannulation seems to be effective in the preventing PEP [23].

As was described above, there are two kinds of meta-analysis-proved agents ef-fective in the preventing or reducing the incidence of PEP, including NAIDs and GTN. And prophylactic pancreatic stent placement was one mechanically effective method, but it is not an easy operation requiring a well-experienced physician, thus it is less applied compared with pharmaceutical agents. With the PEP still being the most dreadful complication after ERCP, if we put the two ways of agents and stent-placement together, maybe it can yield a more effective way to overcome this dreadful complication of ERCP.

More importantly, the adverse effects of using GTN prophylactically before the procedure of ERCP mainly include hypotension and headache and they responded to conventional treatment.

Compared with the previous studies, this systematic meta-analysis had two main differences: First, compared with Bai's and Shao's studies [24,26], this meta-analysis included more RCTs; Second, in addition to the analysis of effect of GTN on the incidence of PEP, this paper also analyzed whether GTN was conducive to the cannulation of bile ducts, and all of other published articles have not conducted any such analysis [24-26].

## Conclusion

In conclusion, our study provides another pooled and updated evidence of the benefits of GTN in preventing post-ERCP pancreatitis and the first pooled evidence of effect of GTN on the cannulation of ducts. This simple, cheap, and safe medication of GTN, especially the sublingual administration, is recommended for prophylactic use before ERCP to prevent pancreatitis and reduce the incidence of post-ERCP pancreatitis. Prophylactic GTN can be used together with other agents such as NAIDs or with pancreatic-stent placement for preventing PEP, maybe yielding a more satisfactory outcome. So widespread prophylactic administration of GTN may significantly reduce the incidence of PEP, resulting in major clinical and economic benefit especially for those centers with a high incidence of PEP, because compared with other prophylactic agents, GTN not only is effective and can be expediently or easily administrated, but is cheap. Given current scepticism regarding the efficacy of any prophylactic medication for ERCP, additional multicentre studies with a large sample are needed for confirming the effectiveness of GTN on preventing PEPs, prior to widespread adoption of this strategy.

## Completing interests

The authors declare that they have no competing interests.

## Authors' contributions

WCH and CB designed the study; CB collected the data, and wrote the manuscript; FT and CB analyzed the available data and assessed the methodological quality of each study in accordance with the criteria by Jadad. All authors read and approved the final manuscript.

## Pre-publication history

The pre-publication history for this paper can be accessed here:

http://www.biomedcentral.com/1471-230X/10/85/prepub
